# Effects of a lower *versus* a higher oxygenation target in intensive care unit patients with chronic obstructive pulmonary disease and acute hypoxaemic respiratory failure: a subgroup analysis of a randomised clinical trial

**DOI:** 10.1016/j.bjao.2024.100281

**Published:** 2024-04-29

**Authors:** Maria B. Nielsen, Thomas L. Klitgaard, Ulla M. Weinreich, Frederik M. Nielsen, Anders Perner, Olav L. Schjørring, Bodil S. Rasmussen

**Affiliations:** 1 Department of Anaesthesia and Intensive Care, Aalborg University Hospital, Aalborg, Denmark; 2 Department of Clinical Medicine, Aalborg University, Aalborg, Denmark; 3 Department of Respiratory Diseases, Aalborg University Hospital, Aalborg, Denmark; 4 Research Unit of Respiratory Diseases, Aalborg University Hospital, Aalborg, Denmark; 5 Department of Intensive Care, Copenhagen University Hospital – Rigshospitalet, Copenhagen, Denmark; 6 Department of Clinical Medicine, University of Copenhagen, Copenhagen, Denmark

**Keywords:** chronic obstructive pulmonary disease, critical care, hyperoxia, hypoxia, intensive care units, oxygen inhalation therapy

## Abstract

**Background:**

Oxygen supplementation is ubiquitous in intensive care unit (ICU) patients with chronic obstructive pulmonary disease (COPD) and acute hypoxaemia, but the optimal oxygenation target has not been established.

**Methods:**

This was a pre-planned subgroup analysis of the Handling Oxygenation Targets in the ICU (HOT-ICU) trial, which allocated patients with acute hypoxaemia to a lower oxygenation target (partial pressure of arterial oxygen [*P*ao_2_] of 8 kPa) *vs* a higher target (*P*ao_2_ of 12 kPa) during ICU admission, for up to 90 days; the allocation was stratified for presence or absence of COPD. Here, we report key outcomes for patients with COPD.

**Results:**

The HOT-ICU trial enrolled 2928 patients of whom 563 had COPD; 277 were allocated to the lower and 286 to the higher oxygenation group. After allocation, the median *P*ao_2_ was 9.1 kPa (inter-quartile range 8.7–9.9) in the lower group *vs* 12.1 kPa (11.2–12.9) in the higher group. Data for arterial carbon dioxide (*P*aco_2_) were available for 497 patients (88%) with no between-group difference in time-weighted average; median *P*aco_2_ 6.0 kPa (5.2–7.2) in the lower group *vs* 6.2 kPa (5.4–7.3) in the higher group. At 90 days, 122/277 patients (44%) in the lower oxygenation group had died *vs* 132/285 patients (46%) in the higher (relative risk 0.98; 95% confidence interval 0.82–1.17; *P*=0.67). No statistically significant differences were found in any secondary outcome.

**Conclusions:**

In ICU patients with COPD and acute hypoxaemia, a lower *vs* a higher oxygenation target did not reduce mortality. There were no between-group differences in *P*aco_2_ or in secondary outcomes.

**Clinical trial registration:**

NCT 03174002, EudraCT number 2017-000632-34.

Chronic obstructive pulmonary disease (COPD) is a major and increasing contributor to morbidity and mortality, and the global COPD burden is projected to increase in coming decades.^1^^,^^2^ Hence, the number of COPD patients admitted to the intensive care unit (ICU) is expected to escalate, both as a result of acute exacerbations of COPD and non-COPD-related acute critical illness. Supplementary oxygen is lifesaving for critically ill patients with acute hypoxaemic respiratory failure and a key component of life support in the ICU. The potential harmful physiological effects of hyperoxia^3^ emphasise a targeted oxygenation approach. However, the evidence on oxygen supplementation in COPD patients admitted to the ICU with acute hypoxaemic respiratory failure is scarce.^4^^,^^5^ In hospitalised COPD patients, the current clinical practice guidelines recommend to target a peripheral oxygen saturation (SpO_2_) of 88% to 92% to minimise the risk of hypercapnic respiratory failure, though a target of 94–98% is suggested in case of normocapnia.^4^^,^^6^^,^^7^ However, oxygen therapy for COPD patients admitted to the ICU are excluded in the current clinical practice guidelines because of limited evidence.^4^^,^^6^ Gomersall and colleagues^8^ randomised 36 COPD patients admitted to the ICU to a partial pressure of arterial oxygen (*P*ao_2_) >6.7 kPa or a *P*ao_2_ >9.3 kPa with no between-group difference in any outcomes. Austin and colleagues^9^ performed a cluster randomised trial of 405 patients with breathlessness suspected to have an acute exacerbation of COPD as judged by paramedics, to receive either 8–10 L of oxygen via face mask (standard care) or titrated oxygen therapy to an SpO_2_ of 88–92%. The authors reported significantly lower mortality (pre-hospital and in-hospital) in the lower oxygenation (titrated) group compared to the standard-of-care higher oxygenation group. However, the trial was conducted in the pre-hospital setting and has a number of significant limitations including a high risk of selection bias as the paramedics, not the patients, were randomised. Additionally, only half of the included patients were subsequently verified as having COPD, and oxygen exposure time was short with an average of 47 min.^9^

The Handling Oxygenation Targets in the ICU (HOT-ICU) trial enrolled 2928 adult patients with acute hypoxaemic respiratory failure.^10^ Patients were randomised within 12 h from ICU admission to targeting a *P*ao_2_ of either 8 kPa or 12 kPa throughout the entire stay in the ICU for up to 90 days, including readmissions. Randomisation was stratified according to the presence or absence of COPD. The primary outcome was all-cause mortality at 90 days, and the trial demonstrated no overall difference between the two oxygenation targets. We present the results of an analysis of a pre-planned subgroup of COPD patients enrolled in the HOT-ICU trial supplemented with detailed analyses of acid–base balances. The aim was to evaluate the benefits and harms of a lower *vs* a higher oxygenation target in COPD patients with acute hypoxaemic respiratory failure in the ICU.

## Methods

### Study design

The HOT-ICU trial was an investigator-initiated, parallel-group, open-label, randomised pragmatic clinical trial, and this is an analysis of a predefined subgroup from the HOT-ICU trial, being all enrolled COPD patients.^11^^,^^12^ This report was prepared in accordance with the Consolidated Standards of Reporting Trials (CONSORT)^13^ and the CONSORT checklist is available in the Supplementary appendix.

### Patients

The criteria for defining a patient with COPD at baseline were: (1) spirometry in stable phase diagnostic of COPD (forced expiratory volume in 1 s [FEV_1_] over forced vital capacity [FVC] ratio <0.70 and an FEV_1_ <80% of predicted) and flow limitations incompletely reversible with inhaled bronchodilators^1^; or (2) medical history of COPD and daily use of inhaled bronchodilators, glucocorticoids, or both. Additional information is presented in the Supplementary appendix.

In this study, we included patients who were at least 18 yr old with COPD acutely admitted to the ICU, requiring at least 10 L of oxygen per minute in an open oxygen supplementation system (including high-flow systems) or a fraction of inspired oxygen (FiO_2_) of at least 0.50 in a closed system (invasive or noninvasive mechanical ventilation, or continuous positive airway pressure by mask), with expected requirement of supplementary oxygen in the ICU for at least 24 h, and having an arterial cannula for frequent *P*ao_2_ monitoring. Patients were excluded if they could not be recruited within 12 h of ICU admission, if they received home oxygen or chronic mechanical ventilation, or if consent could not be obtained; additional details on inclusion and exclusion criteria are presented in the Supplementary appendix.

### Randomisation and masking

Randomisation was conducted using a computer-generated concealed assignment sequence with permuted blocks of varying sizes and was stratified according to trial site, presence or absence of COPD, and presence or absence of active haematological malignancy. Trial group assignment was not masked to patients, relatives, clinicians, or research staff.

### Procedures

Patients were randomly allocated 1:1 to receive oxygen therapy targeting a *P*ao_2_ of 8 kPa (lower oxygenation group) or a *P*ao_2_ of 12 kPa (higher oxygenation group) during ICU admission until 90 days post-allocation, including any ICU readmissions. The oxygenation target was achieved by adjusting the FiO_2_. Given the pragmatic trial design, all other treatments, including choice of oxygen delivery device and ventilator settings, were at the discretion of the treating clinician.^11^ The highest and lowest measurements of *P*ao_2_, with concomitant measures of arterial oxygen saturations (SaO_2_) and fractions of inspired oxygen (FiO_2_) were registered in predefined 12-h intervals during ICU admission. Supplementary data for all arterial blood gas analyses were obtained from all Danish patients (Radiometer Medical ApS, Copenhagen, Denmark).

### Outcomes

The primary outcome was 90-day all-cause mortality. Secondary outcomes were: ‘days alive without life support’ (mechanical ventilation, renal replacement therapy, or vasopressor or inotrope infusion); ‘days alive and out of hospital’; ‘proportion of patients with one or more serious adverse events (SAE) in the ICU’ (new episodes of shock, myocardial ischaemia, cerebral ischaemia, or intestinal ischaemia) all within 90 days; and 1-yr all-cause mortality. Additional details on outcome definitions are presented in the Supplementary appendix.

### Statistical analyses

No sample size estimation was performed as this was a subgroup analysis. Analyses were conducted according to the HOT-ICU statistical analysis plan and the intention-to-treat principle in patients with COPD at baseline, for whom there was consent to the use of data.^12^ We compared 90-day mortality using a generalised linear model with binomial error distribution and a log-link to produce a relative risk (RR), and an identity link to produce a risk difference (RD). These mortality analyses were adjusted for trial site and presence or absence of active haematological malignancy. One-year all-cause mortality and the proportion of patients with one or more SAEs in the ICU were analysed similarly to the 90-day mortality, though with adjustment for trial site only because of non-convergence in the statistical models. Results are reported with 95% confidence intervals (CI).

We performed a secondary analysis of 90-day mortality with additional adjustments for important baseline variables being age, presence or absence of active metastatic cancer, admission type, and Sequential Organ Failure Assessment (SOFA) score, using logistic regression. Because of non-parametric data distribution, we analysed the secondary outcomes days alive without life support and days alive out of hospital using the van Elteren-test, adjusting for trial site only. Mortality analyses were complemented with Kaplan–Meier plots. Median values for the entire 90-day intervention period for *P*ao_2_, FiO_2_, and SaO_2_ were calculated from the registered 12-h highest and lowest *P*ao_2_ with concomitant values of FiO_2_ and SaO_2_. Graphs of daily values are also presented. Time-weighted averages were calculated for all arterial blood gas analyses obtained during ICU stay from the Danish patients focusing on *P*ao_2_, SaO_2_, partial pressure of arterial carbon dioxide (*P*aco_2_), pH, and standard bicarbonate. Median values for the entire 90-day period are presented. Results are reported as median patient time-weighted averages for each variable for the entire intervention period in each intervention group, supplemented with graphs of daily patient time-weighted averages. We considered *P*-values <0.05 statistically significant. No imputations for missing values were performed. All analyses were performed using STATA statistical software, release 17 (StataNordic, Metrika Consulting AB, Birger Jarlsgatan 2, 5th floor, 114 34 Stockholm, Sweden). The authors MBN, TLK, FMN, OLS, and BSR had full access to all study data. The final decision to submit for publication was made by the corresponding author. All authors vouch for the completeness and accuracy of the reported data.

## Results

### Trial population

**Fig 1:**
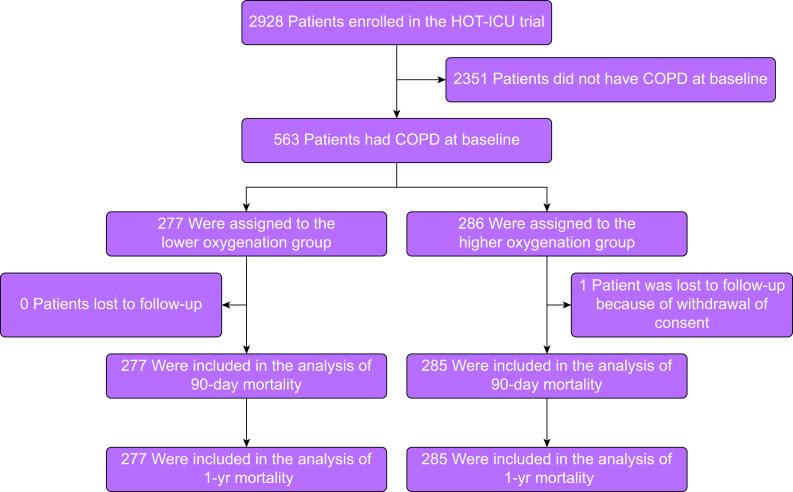
Patient flow. COPD, chronic obstructive pulmonary disease; HOT-ICU, Handling Oxygenation Targets in the Intensive Care Unit.

Patients were recruited into the HOT-ICU trial from 20 June 2017 to 3 August 2020. Of the 2928 patients randomised, 563 patients had COPD at baseline (19%). These were enrolled at 29 sites in Denmark, Switzerland, Finland, the Netherlands, Norway, and the UK; 277 were assigned to the lower oxygenation group and 286 to the higher oxygenation group. One patient in the higher oxygenation group was lost to follow-up for all outcomes because consent was withdrawn (Fig. 1). Baseline characteristics were similar between the two groups, except for *P*ao_2_ and the proportion of patients with cardiac arrest (Table 1). Patients included in this sub-study presented with higher levels of *P*aco_2_ and standard bicarbonate, and lower pH values compared with the remaining HOT-ICU cohort. In addition, chronic heart failure was more prevalent in COPD patients, whilst presence of active haematological malignancy was more prevalent in patients without COPD (Supplementary Table S1).

**Table 1:** Baseline characteristics. Values are medians unless stated otherwise. ARDS, acute respiratory distress syndrome; CPAP, continuous positive airway pressure; FiO_2_, fraction of inspired oxygen; ICU, intensive care unit; IQR, inter-quartile range; NE, norepinephrine; NIV, noninvasive ventilation; *P*aco_2_, partial pressure of arterial carbon dioxide; *P*ao_2_, partial pressure of arterial oxygen; SaO_2_, oxygen saturation; SOFA, Sequential Organ Failure Assessment. ∗Values for SaO_2_ were missing for 11 patients in the lower and 14 patients in the higher oxygenation group as this parameter was not available at one trial site. ^†^*P*aco_2_, pH, and standard bicarbonate are based on arterial blood gases from 497 Danish patients with COPD.^‡^ FiO_2_ in open systems are estimated using standardised conversion tables (Supplementary Table S5). ^¶^In patients receiving norepinephrine. ^§^SOFA scores range from 0 to 24, with higher scores indicating more severe organ failure.

Characteristic	Lower oxygenation group (*n*=277)	Higher oxygenation group (*n*=286)
Age (IQR)	72 (65–77)	71 (63–77)
Male sex, *n* (%)	177 (63.9)	165 (57.7)
Time from hospital admission to randomisation (days) (IQR)	1 (0–4)	1 (0–4)
Time from ICU admission to randomisation (h) (IQR)	3.4 (1.4–7.0)	3.4 (1.5–7.3)
Coexisting illness, *n* (%)		
Ischaemic heart disease	45 (16.3)	45 (15.7)
Chronic heart failure	40 (14.4)	45 (15.7)
Active metastatic cancer	5 (1.8)	12 (4.2)
Long-term dialysis	1 (0.4)	5 (1.8)
Active haematological malignancy	8 (2.9)	8 (2.8)
Type of admission, *n* (%)		
Medical	248 (89.5)	242 (84.6)
Elective surgery	4 (1.4)	7 (2.5)
Emergency surgery	25 (9.0)	37 (12.9)
Acute illness, *n* (%)		
Pneumonia	173 (62.5)	171 (59.8)
Multiple trauma	2 (0.7)	6 (2.1)
Haemorrhagic or ischaemic stroke	1 (0.4)	5 (1.8)
Traumatic brain injury	1 (0.4)	2 (0.7)
Myocardial infarction	14 (5.1)	16 (5.6)
Intestinal ischemia	6 (2.2)	5 (1.8)
Cardiac arrest	19 (6.9)	37 (12.9)
ARDS	20 (7.2)	24 (8.4)
Invasive ventilation		
Patients, *n* (%)	156 (56.3)	171 (59.8)
Tidal volume (ml) (IQR)	500 (430–564)	503 (421–584)
End-expiratory pressure (cm H_2_O) (IQR)	8 (7–10)	8 (7–10)
Peak pressure (cm H_2_O) (IQR)	25 (22–30)	26 (21–31)
Noninvasive ventilation or CPAP		
Patients, *n* (%)	53 (19.1)	51 (17.8)
End-expiratory pressure (cm H_2_O) (IQR)	7 (5–8)	8 (6–9)
Open system, *n* (%)	68 (24.6)	64 (22.4)
*P*ao_2_ (kPa) (IQR)	10.7 (8.9–13.0)	10.1 (8.6–12.5)
SaO_2_ (%) (IQR)∗	95 (91–97)	94 (90–97)
*P*aco_2_ (kPa) (IQR)^†^	6.3 (5.4–8.4)	6.5 (5.4–8.1)
pH (IQR)^†^	7.31 (7.24–7.38)	7.31 (7.24–7.36)
Standard bicarbonate (mmol L^−1^) (IQR)^†^	22.7 (19.7–26.0)	22.3 (19.7–25.7)
FiO_2_, fraction (IQR)^‡^	0.70 (0.50–0.80)	0.60 (0.60–0.80)
*P*ao_2_:FiO_2_ ratio (IQR)		
In all systems	16.6 (12.9–21.5)	15.8 (12.9–21.1)
Invasive ventilation	18.3 (13.9–22.5)	17.2 (13.0–23.0)
NIV/CPAP	16.5 (13.7–21.7)	16.2 (13.5–19.4)
Open systems	13.5 (11.4–18.0)	14.1 (11.9–18.0)
Lactate concentration (mmol L^−1^) (IQR)	1.6 (1.0–2.8)	1.6 (1.1–2.7)
Lowest mean arterial pressure (mm Hg) (IQR)	59 (50–69)	57 (47–67)
Use of inotropes, *n* (%)	5 (1.8)	4 (1.4)
Use of vasopressors		
Patients, *n* (%)	139 (50.2)	154 (53.9)
Highest dose of NE (μg kg^−1^) min^−1^ (IQR)^¶^	0.2 (0.1–0.4)	0.2 (0.1–0.4)
SOFA score (IQR)^§^	7 (5–9)	7 (5–10)

### Oxygenation and ICU interventions

**Fig 2:**
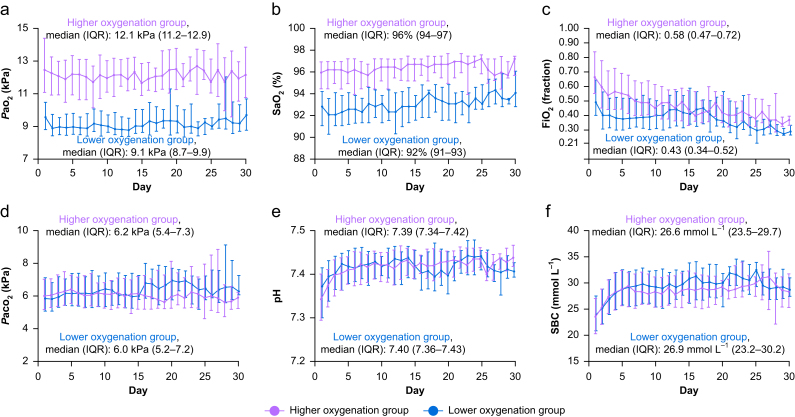
Oxygenation and arterial blood gas data until 30 days after randomisation. Group medians with inter-quartile ranges (IQR) are calculated for the 90-day intervention period. Data for the number of patients contributing with data are reported in Supplementary Figure S9 and Supplementary Table S4 in the Supplementary Appendix. Data on the full 90-day intervention period are reported in Supplementary Figures S1–S8. (A)–(C) Oxygenation data until 30 days after randomisation, for 563 patients with chronic obstructive pulmonary disease (COPD) enrolled in the Handling Oxygenation targets in the Intensive Care Unit (HOT-ICU) trial. Values are medians of daily patient-means with IQR. Daily patient-means were calculated from the lowest and highest *P*ao_2_ in 12-h intervals, with concomitant values for SaO_2_ and FiO_2_. SaO_2_ data are missing for 25 patients (11 in the lower oxygenation group and 14 in the higher oxygenation group) as this analysis was unavailable at one trial site. (D)–(F) Arterial blood gas data until 30 days after randomisation, based on the 497 Danish COPD patients enrolled in the HOT-ICU. Values are daily medians of patients' time-weighted averages with IQR. FiO_2_, fraction of inspired oxygen; *P*aco_2_, partial pressure of arterial carbon dioxide; *P*ao_2_, partial pressure of arterial oxygen; SaO_2_, oxygen saturation; SBC, standard bicarbonate.

Data for the first 30 days, based on the 12-h highest and lowest *P*ao_2_ measurements, with concomitant FiO_2_ and SaO_2_ values, are presented for the entire COPD cohort, whilst data based on calculations of time-weighted averages of *P*aco_2_, pH, and standard bicarbonate are presented for the 497 (88%) Danish patients only (Fig. 2). Data on oxygenation and arterial blood gas variables for the entire 90-day period are presented in Supplementary Figures S1–S8. We found clear separation in all oxygenation variables (Fig. 2 and Supplementary Figs S1–S5).

On average, six daily blood gases were drawn from each patient during the intervention period in both groups (Supplementary Tables S2 and S3). However, we only mandated registration of data corresponding to the highest and lowest *P*ao_2_ measurement in predefined 12-h intervals. *Post hoc* we collected data on all arterial blood gas analyses for the 497 Danish COPD patients, comprising a total of 27 045 samples conducted during ICU stay (12 715 from the lower oxygenation group and 14 272 from the higher oxygenation group). We found no between-group differences for *P*aco_2_, pH, or bicarbonate in the 497 Danish patients (Fig. 2 and Supplementary Figs S6–S8). Data on the number of patients contributing with oxygenation data are presented in Supplementary Table S4 and Supplementary Figures S9 and S10. Standardised conversion tables for FiO_2_ in open systems are presented in Supplementary Table S5.

We found no between-group differences in ICU treatment, including the need for invasive mechanical ventilation, except for higher end-expiratory pressures during invasive and noninvasive ventilation in the higher compared with the lower oxygenation group (Supplementary Table S2). ICU treatment was comparable between COPD patients and the remaining HOT-ICU cohort except for use of noninvasive ventilation (29% of COPD patients *vs* 21% of non-COPD patients) and renal replacement therapy (12% of COPD patients *vs* 23% of non-COPD patients) (Supplementary Table S3).

### Outcomes

**Fig 3:**
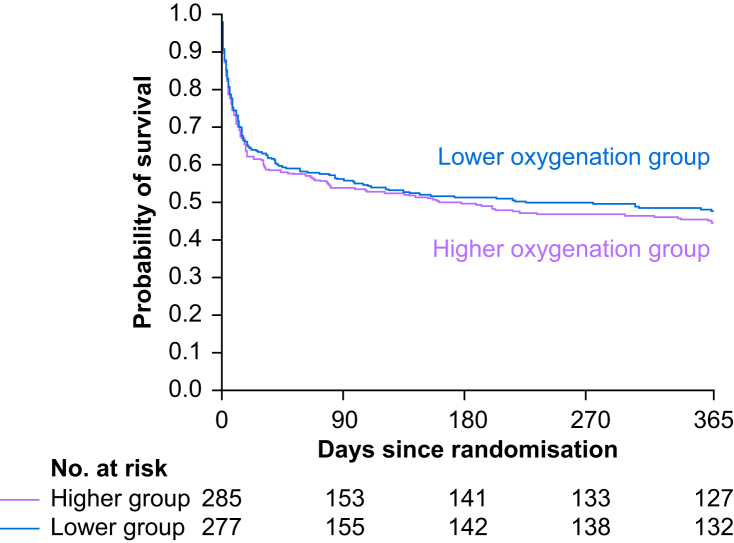
Kaplan–Meier estimates of survival. Administratively censored 1 yr post randomisation. Hazard ratios (HR) from Cox proportional-hazards model adjusted for trial site and presence or absence of active haematological malignancy; 90-day all-cause mortality HR: 0.95 (95% confidence interval 0.73–1.19, *P*=0.58); 1-yr all-cause mortality HR: 0.94 (95% confidence interval 0.75–1.18, *P*=0.69).

At 90 days, 122 of 277 patients (44%) in the lower oxygenation group and 132 of 285 patients (46%) in the higher oxygenation group had died (RR 0.98; 95% CI 0.82–1.17; *P*=0.83). At 1 yr, 145 of 277 (52%) patients in the lower oxygenation group, and 159 of 285 (56%) patients in the higher oxygenation group had died (RR 0.97; 95% CI 0.83–1.13; *P*=0.67) (Table 2, Fig. 3). The all-cause mortality of COPD patients was higher compared with the remaining HOT-ICU cohort at both 90 days (45 *vs* 42%) and 1 yr (54% *vs* 48%). We found no significant interaction in the entire HOT-ICU intention-to-treat population between the oxygenation target allocation and the presence or absence of COPD for neither 90-day (*P*=0.58) nor 1-yr all-cause mortality (*P*=0.61). There were no statistically significant differences between the two groups for days alive without life support, days alive out of hospital, or proportions of patients with one or more SAEs in the ICU (Table 2). Also, we found that the two groups were treated similarly with respect to the use of both invasive and noninvasive mechanical ventilation (Supplementary Table S2).

**Table 2:** Outcomes. CI, confidence interval; ICU, intensive care unit; IQR, inter-quartile range; SAE, serious adverse event. ∗Data regarding primary outcome were missing for one patient in the higher oxygenation group. ^†^Stratification variables: trial site and active haematological malignancy for 90-day mortality. ^‡^Baseline characteristics: age; active metastatic cancer; type of admission (medical, elective surgical, or acute surgical); Sequential Organ Failure Assessment score ranging from 0 to 24, with higher values indicating more severe organ failure. ^¶^Life support was defined as any use of mechanical ventilation (invasive ventilation, noninvasive ventilation or non-intermittent continuous positive airway pressure), circulatory support (vasopressor or inotropic infusion), or renal replacement therapy.

	Lower oxygenation group	Higher oxygenation group	Relative risk (95% CI)	Risk difference (95% CI)	Odds ratio (95% CI)	*P*-value
Primary outcome∗						
90-Day all-cause mortality, *n*/*n*-total (%)	122/277 (44.0)	132/285 (46.3)				
Adjusted for stratification variables^†^			0.98 (0.82–1.17)	−2.59 (−10.70 to 5.51)		0.83
Adjusted for stratification variables^†^ and baseline characteristics^‡^					0.92 (0.64–1.32)	0.65
Secondary outcomes						
1-Yr all-cause mortality, *n* (%)	145/277 (52.3)	159/285 (55.8)				
Adjusted for site			0.97 (0.83–1.13)	−3.21 (−11.35 to 4.93)		0.67
Median number of days alive without life support in 90 days (IQR)^¶^	78 (1–87)	70 (0–86)				0.24
Mechanical ventilation	79 (1–87)	70 (0–87)				
Circulatory support	83 (3–88)	78 (3–88)				
Renal replacement therapy	90 (7–90)	85 (7–90)				
Median number of days alive out of hospital in 90 days (IQR)	45 (0–74)	36 (0–71)				0.60
Number of patients with one or more SAE in the ICU within 90 days, *n*/*n*-total (%)	110/277 (39.7)	111/285 (38.8)	1.03 (0.84–1.26)	0.89 (−7.16 to 8.93)		0.77
New shock	101 (36.5)	106 (37.1)				
New myocardial infarction	3 (1.1)	1 (0.4)				
New ischemic stroke	5 (1.8)	5 (1.8)				
New intestinal ischemia	8 (2.9)	7 (2.5)

## Discussion

In this analysis of a pre-planned subgroup of COPD patients with severe hypoxaemic respiratory failure enrolled in the HOT-ICU trial, a lower oxygenation target did not reduce short- or long-term mortality compared with a higher oxygenation target. Also, there were no statistically significant differences in any secondary outcomes.

The mortality rates in our patients were higher than reported in recent observational studies in patients with COPD admitted to the ICU for either acute exacerbations^14^, ^15^, ^16^ or for non-COPD reasons.^17^ This likely stems from the fact that our COPD cohort presented with severe hypoxaemic respiratory failure at inclusion. Our findings agree with those reported in the small-scale randomised trial by Gomersall and colleagues^8^ whereas we were unable to demonstrate a mortality reduction as suggested by Austin and colleagues.^9^

In hospitalised patients with COPD, or other chronic diseases with an increased risk of hypercapnia, targeting an SpO_2_ of 88% to 92% has been entrenched practice for years. This appears to be based primarily on the trial by Austin and colleagues,^9^ and on estimates that 20–50% of COPD patients with acute exacerbations are at risk of carbon dioxide retention if given excessive supplementary oxygen therapy.^4^ However, there are too few data on which to base recommendations for oxygenation targets in hypoxaemic COPD patients in the ICU.^4^ Ours is the largest randomised study to date of acutely ill COPD patients admitted to an ICU with hypoxaemic respiratory failure comparing a lower *vs* a higher oxygenation target. Previous large-scale, randomised trials on oxygenation strategies in the ICU have provided minimal evidence^5^; two trials excluded COPD patients altogether,^18^^,^^19^ two excluded severe or exacerbated COPD,^20^^,^^21^ and two included COPD patients but were both prematurely stopped.^22^^,^^23^ A recent cluster randomised trial included COPD patients but has not reported any results specific for this subset of patients.^24^

COPD patients constituted approximately one-fifth of the entire HOT-ICU cohort, which supports the notion that such patients contribute substantially to the total burden of ICU patients with acute hypoxaemic respiratory failure. This percentage could be even higher in clinical practice as clinicians report targeting a lower oxygenation level in COPD patients,^25^ despite a lack of evidence,^4^ thus not fulfilling the required FiO_2_ for inclusion into the present trial. Arterial blood gas analyses obtained from the Danish patients, constituting 88% of all COPD patients included in the HOT-ICU trial, showed slightly higher *P*aco_2_ and lower pH at baseline in COPD patients compared with those without COPD, which suggests that there were more patients with respiratory acidosis in the COPD cohort. We expected that a higher oxygenation target would increase the use of mechanical ventilation in the COPD cohort because of the assumed risk of CO_2_ retention. Interestingly, we found that patients in the two groups received a similar number of days of mechanical ventilation (both invasive and noninvasive), and comparable 90-day time-weighted values for *P*aco_2_, pH, and standard bicarbonate. Thus, the higher oxygenation target had little or no impact on the risk of respiratory acidosis during the ICU stay, on tracheal intubation rates, or on weaning from mechanical ventilation.

### Strengths of this study

Several strengths of this sub-study are worth emphasising. This was a stratified, pre-planned subpopulation of patients with COPD, with interventions representative for ICU patients and individual patient randomisation with targeted oxygenation for up to 90 days, enabling valid inference.^26^ Also, all patients had an arterial line and participating sites had access to bedside arterial blood gas analysers. Collection of all arterial blood gas analyses conducted in Danish COPD patients throughout the intervention period provided a unique opportunity for detailed analyses of acid–base disturbances. Additionally, the international enrolment and pragmatic trial design increased the external validity.^10^ Lastly, we achieved excellent between-group separation of *P*ao_2_, SaO_2_, and FiO_2_ while treating patients equally regarding other ICU interventions.

### Limitations of the study

As this study included COPD patients with acute hypoxaemic respiratory failure, the results may not be transferable to patients with isolated or severe hypercapnic respiratory failure (with or without hypoxaemia) or admitted to the ICU for non-COPD-related illness. The use of standardised conversion tables for FiO_2_ in open systems do not necessarily reflect the true FiO_2_, as this is highly dependent on the individual patient condition. Finally, although the subgroup was pre-planned, the results should be interpreted with caution.

### Conclusion

In this subgroup analysis of ICU patients with COPD and acute hypoxaemic respiratory failure, a lower oxygenation target did not reduce mortality compared with a higher oxygenation target. There were no differences between the groups in secondary outcomes nor in *P*aco_2_ levels during the ICU stay.

## Authors’ contributions

Conducted all analyses: MBN, TLK, FMN.

Wrote the first draft: MBN.

Critically revised the first draft: all authors.

Designed the HOT-ICU trial: BSR, OLS, AP.

Sponsor and principal investigator of the HOT-ICU trial: BSR.

Coordinating investigators of the HOT-ICU trial: OLS, TLK.

Detailed author contributions for the complete trial were presented in the primary trial report.^17^

## Declarations of interest

The Department of Intensive Care at Rigshospitalet has received funding for other projects from the Novo Nordisk Foundation, Pfizer, Sygeforsikringen ‘danmark’, and Fresenius Kabi and does contract research for AM Pharma. UMW has received fees, including transportation reimbursement for: congress participation: Orion Pharma; advisory boards: TEVA, Astra Zeneca, Chiesi, and Novartis; speaker's fees: AstraZeneca, Chiesi, Novartis, Boehringer Ingelheim, GlaxoSmithKline (GSK), Orion Pharma, Fisher & Paykel Healthcare, and ResMed; writer's fee: AstraZeneca; pharma-initiated research: AstraZeneca, GSK, Novartis, Ins-Med, Sanofi, Novartis, Boehringer Ingelheim, and Genetech; Research grant: Fisher & Paykel Healthcare and Liita Healthcare.

## Ethics statement

Obtainment of informed consent and use of data followed national regulations. The HOT-ICU trial was approved by the Danish Medicines Agency (AAUH-ICU-01, EudraCT no. 2017-000632-34); the Committee on Health Research Ethics in the North Denmark Region (N-20170015); the Danish Data Protection Agency (2008-58-0028), and all required authorities in the participating countries, and registered prospectively at ClinicalTrials.gov (NCT03174002). The HOT-ICU trial protocol, statistical analysis plan, and results in the main cohort are available elsewhere.^10^, ^11^, ^12^^,^^27^

## Data sharing statement

Complete deidentified patient data set collected during the HOT-ICU trial will be shared beginning 2 yr after 8 April 2021, with no end date. Data will be available to researchers who provide a methodologically sound proposal for the purposes of achieving specified aims. The proposals will be reviewed by the HOT-ICU trial Management Committee. To gain access, the researchers will need to sign a data access agreement and to confirm that data will only be used for the agreed purpose for which access was granted. Request for data must be sent to the primary investigator and HOT-ICU sponsor via email: bodil.steen.rasmussen@rn.dk.

## Transparency

All authors affirm that the manuscript is an honest, accurate, and transparent account of the study being reported; that no important aspects of the study have been omitted; and that any discrepancies from the study as originally planned (and, if relevant, registered) have been explained.

## Funding

The HOT-ICU trial was funded by a grant from Innovation Fund Denmark (4108-00011A) and supported by Aalborg University Hospital, the Regions of Denmark (EMN-2017-00901 and EMN-2019-01055), the Obel Family Foundation (25457), the Danish Society of Anaesthesiology and Intensive Care Medicine, and the Intensive Care Symposium Hindsgavl. No additional funding was provided for this sub-study. Funders of this study had no role in the design, collection, analysis, interpretation of data, or writing of this report.
