# Supplementary material for: Effects of a lower versus a higher oxygenation target in intensive care unit patients with chronic obstructive pulmonary disease and acute hypoxaemic respiratory failure: a subgroup analysis of a randomised clinical trial
Source: BJA Open. 2024 Apr 29;10:100281. doi: 10.1016/j.bjao.2024.100281 (PMC11070685; doi:10.1016/j.bjao.2024.100281)
Supplement: Multimedia component 1 [file mmc1.docx]

**Effects of lower vs higher oxygenation targets in intensive care unit patients with chronic obstructive pulmonary disease and acute hypoxaemic respiratory failure: a subgroup analysis of a randomised clinical trial**

Maria Brun Nielsen,^1,2^ Thomas Lass Klitgaard,^1^ Ulla Møller Weinreich,^2,3,4^ Frederik Mølgaard Nielsen,^1,2^

Anders Perner,^5,6^ Olav Lilleholt Schjørring,^1,2^ Bodil Steen Rasmussen^1,2,*^

^1^Department of Anaesthesia and Intensive Care, Aalborg University Hospital, Aalborg, Denmark

^2^Department of Clinical Medicine, Aalborg University, Aalborg, Denmark

^3^Department of Respiratory Diseases, Aalborg University Hospital, Aalborg, Denmark

^4^Research Unit of Respiratory Diseases, Aalborg University Hospital, Aalborg, Denmark

^5^Department of Intensive Care, Copenhagen University Hospital – Rigshospitalet, Copenhagen Denmark

^6^Department of Clinical Medicine, University of Copenhagen, Copenhagen, Denmark

*Corresponding author. E-mail: [bodil.steen.rasmussen@rn.dk](mailto:bodil.steen.rasmussen@rn.dk)

Supplementary appendix

**Table of content**

[Inclusion and exclusion criteria for the HOT-ICU trial 2](#_Toc160788204)

[Definition of chronic obstructive pulmonary disease in the HOT-ICU trial 4](#_Toc160788205)

[Outcome definitions 5](#_Toc160788206)

[Figure S1. Daily patient-mean PaO_2_ 6](#_Toc160788207)

[Figure S2. Daily patient-mean SaO_2_ 7](#_Toc160788208)

[Figure S3. Daily patient-mean FiO_2_ 8](#_Toc160788209)

[Figure S4. Time-weighted average PaO_2_ 9](#_Toc160788210)

[Figure S5. Time-weighted average SaO_2_ 10](#_Toc160788211)

[Figure S6. Time-weighted average PaCO_2_ 11](#_Toc160788212)

[Figure S7. Time-weighted average pH 12](#_Toc160788213)

[Figure S8. Time-weighted average SBC 13](#_Toc160788214)

[Figure S9. Number of patients providing arterial blood gas data for the full arterial blood gas analyses 14](#_Toc160788215)

[Figure S10. Number of arterial blood gases per day for the full arterial blood gas analyses 15](#_Toc160788216)

[Table S1. Baseline Characteristics of patients with and without COPD 16](#_Toc160788217)

[Table S2. Intensive care unit treatment parameters in patients with COPD 18](#_Toc160788218)

[Table S3. Intensive care unit treatment parameters in patients with and without COPD 19](#_Toc160788219)

[Table S4. Number of COPD patients providing data on oxygenation in the HOT-ICU trial 20](#_Toc160788220)

[Table S5. Fraction of inspired oxygen (FiO_2_) conversion tables for open systems 21](#_Toc160788221)

[CONSORT 2010 checklist 22](#_Toc160788222)

[References 25](#_Toc160788223)

# Inclusion and exclusion criteria for the HOT-ICU trial

**Inclusion criteria**

- Acute admission to the intensive care unit (ICU) (a non-planned admission, which does not include planned recovery after surgery or similar planned admissions. ICU admission does not include admissions to semi-intensive care units, intermediate care units or similar high-dependency units)
- ≥18 years (the age of the patient in whole years at the time of randomisation. The age should be calculated from the date of birth)
- Supplemental oxygen:
- ≥10 litres oxygen per minute in an open system irrespective of any flow of atmospheric air including high-flow systems
- FiO_2_ ≥0.50 in a closed system (invasive mechanical ventilation, or non-invasive (mask or helmet) ventilation, or continuous positive airway pressure (CPAP) systems (mask or helmet))
- Expected duration of supplemental oxygen of at least 24 hours in the ICU (the treating clinician estimates that the patient will need supplementary oxygen for more than 24 hours and remain in the ICU for 24 hours. When in doubt about this forecast, the patient should be enrolled)
- Arterial line for PaO_2_ monitoring in place (a functioning catheter for the sampling of arterial blood must be in place at the time of enrolment)

**Exclusion criteria**

- Cannot be randomised within 12 hours of ICU admission: defined as 12 full hours from the time of the present ICU admission; if the patient is transferred from another ICU, the 12 hours will count from the time of the admission to the first ICU
- Receives chronic mechanical ventilation (invasive mechanical ventilation, continuous non-invasive ventilation or continuous mask-CPAP) for any reason. Nocturnal CPAP or non-invasive ventilation due to sleep apnoea and/or obesity hypoventilation syndrome is not regarded as chronic mechanical ventilation
- Use of supplementary oxygen at home (supplementary oxygen given through nasal cannula, mask or tracheostomy on a regular daily basis independent of whether it is continuous, in daytime or nocturnal)
- Previous treatment with bleomycin (any history of bleomycin treatment documented in the patient charts)
- Organ transplant (any kind of solid organ transplant planned or performed during current hospitalisation)
- Withdrawal from active therapy or brain death deemed imminent (clinicians or investigators judge that withdrawal from active therapy or brain death is likely within a few hours)
- Pregnancy confirmed by a positive urine or plasma human chorionic gonadotropin (hCG) (in women <50 years of age, a negative hCG-test must be present before enrolment)
- Carbon monoxide poisoning (confirmed by an arterial or venous blood carboxyhaemoglobin >3% for non-smokers or >10% for active smokers during current hospitalisation)
- Cyanide poisoning (if suspected by the clinicians during current hospitalisation and documented in the patient charts)
- Paraquat poisoning (if suspected by the clinicians during current hospitalisation and documented in the patient charts)
- Methemoglobinemia (a confirmed arterial or venous blood methaemoglobin >8% during current hospitalisation)
- Sickle cell disease (any history of sickle cell disorder documented in patient charts or the presence of haemoglobin S (HbS) in an arterial or a venous blood sample)
- Any condition expected to involve the use of hyperbaric oxygen (HBO) treatment (e.g. necrotising soft tissue infection in sites using HBO for this condition)
- Consent not obtainable according to national regulations (patients where the clinician or investigator is unable to obtain the necessary consent before inclusion of the patient according to the national regulations)
- Previously randomised into the HOT-ICU trial (previously screened patients may be eligible if they were not randomised)

# Definition of chronic obstructive pulmonary disease in the HOT-ICU trial

One of the following two criteria:

• Conducted spirometry in stable phase that is diagnostic of chronic obstructive pulmonary disease (COPD): A forced expiratory volume in one second/forced vital capacity (FEV_1_/FVC) ratio less than 0.7 or less than the lower limit of normal AND an FEV_1_ less than 80 percent of predicted AND flow limitations must be incompletely reversible after the administration of an inhaled bronchodilator.

• Anamnestic COPD AND daily use of inhaled β_2_-adrenergic and/or anticholinergic bronchodilators and/or inhaled glucocorticoids including albuterol, levalbuterol, salmeterol, formoterol, arformoterol, indacaterol, vilanterol, olodaterol, tiotropium, aclidinium, umeclidinium, glycopyrronium, budesonide, and fluticasone.

# Outcome definitions

**Primary outcome:**

- 90-day mortality: death from any cause within 90 days following the day of randomisation

**Secondary outcomes:**

- 1-year mortality: death from any cause within one year following the day of randomisation
- Proportion of patients with one or more of the following serious adverse events in the ICU after randomisation:
- *New episode of shock:* plasma lactate concentration (p-lactate) >2.0 mmol/L and the use of continuous vasopressor or inotropic treatment on any day in the ICU in participants who did not have shock at baseline (p-lactate ≤2.0 mmol/L or no use of continuous vasopressor or inotropic treatment) or who were shock-free on any of the previous post-randomisation days. Shock-free in any day is defined as highest daily p-lactate ≤2.0 mmol/L and no use of vasopressor or inotropic treatment
- *New myocardial ischaemia:* ST-elevation myocardial infarction, non-ST-elevation myocardial infarction, or unstable angina pectoris according to the criteria of the clinical condition in question, and receiving treatment because of this (reperfusion strategies, or initiation of or increased antithrombotic treatment)
- *New ischaemic stroke:* cerebral computerised tomography (CT) or magnetic resonance imaging (MRI) scan conducted on this day with signs of new ischaemic stroke. Radiographic signs of old infarctions estimated to have occurred before randomisation are not considered new ischaemic stroke. Radiographically diagnosed diffuse anoxic brain injury after pre-randomisation cardiac arrest is not considered new ischaemic stroke
- *New intestinal ischaemia:* onset of gastric, mesenteric, or colonic ischaemia on this day, verified by exploratory or diagnostic abdominal surgery, endoscopic procedures, or on CT or MRI angiography
- Days alive without life support: days alive with the use of mechanical ventilation (invasive mechanical ventilation, non-invasive ventilation, or non-intermittent CPAP), circulatory support (continuous infusion of vasopressor or inotrope), or any form of renal replacement therapy (in patients receiving intermittent renal replacement therapy days between treatments are included as being with the use of renal replacement therapy) within 90 days after randomisation
- Days alive and out of hospital: days alive and out of hospital within 90 days after randomisation

# **Figure S1. Daily patient-mean PaO_2_**


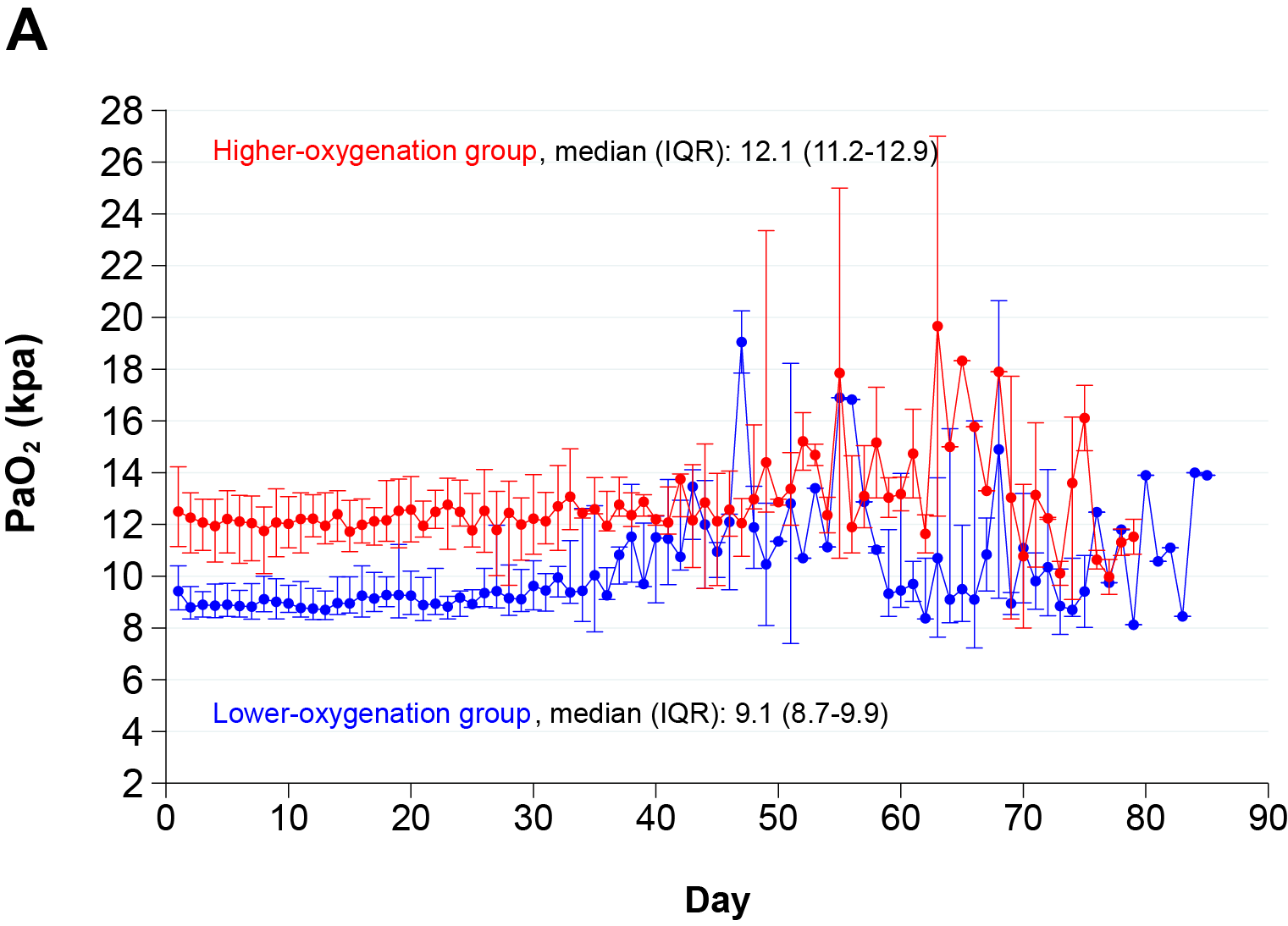


Median values of daily patient-means of partial pressure of arterial oxygen (PaO_2_) stratified according to oxygenation target allocation for the 90-day intervention period in patients with chronic obstructive pulmonary disease. Daily patient-means were calculated from the lowest and highest PaO_2_ in 12-hour intervals. Bars represent interquartile ranges (IQR).

# **Figure S2. Daily patient-mean SaO_2_**


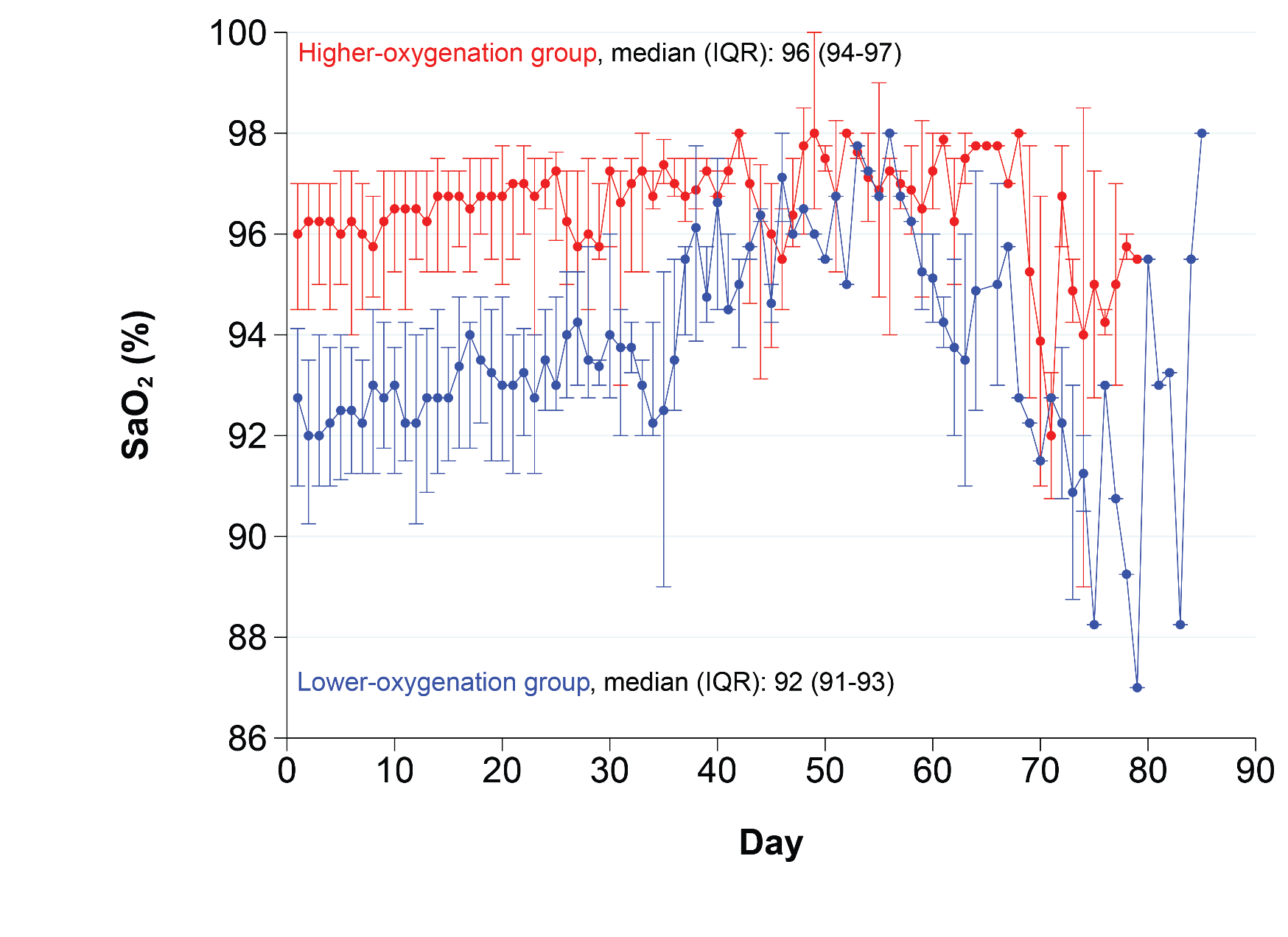


Median values of daily patient-means of arterial oxygen saturation (SaO_2_) stratified according to oxygenation target allocation for the 90-day intervention period in patients with chronic obstructive pulmonary disease. Daily patient-means were calculated from the registered values corresponding to the 12-hour highest and lowest partial pressure of arterial oxygen. Bars represent interquartile ranges (IQR). Data for arterial oxygen saturation (SaO_2_) were not available for 14 patients in the higher oxygenation group and for 11 patients in the lower oxygenation group because this parameter was not available at one site.

# **Figure S3. Daily patient-mean FiO_2_**

**
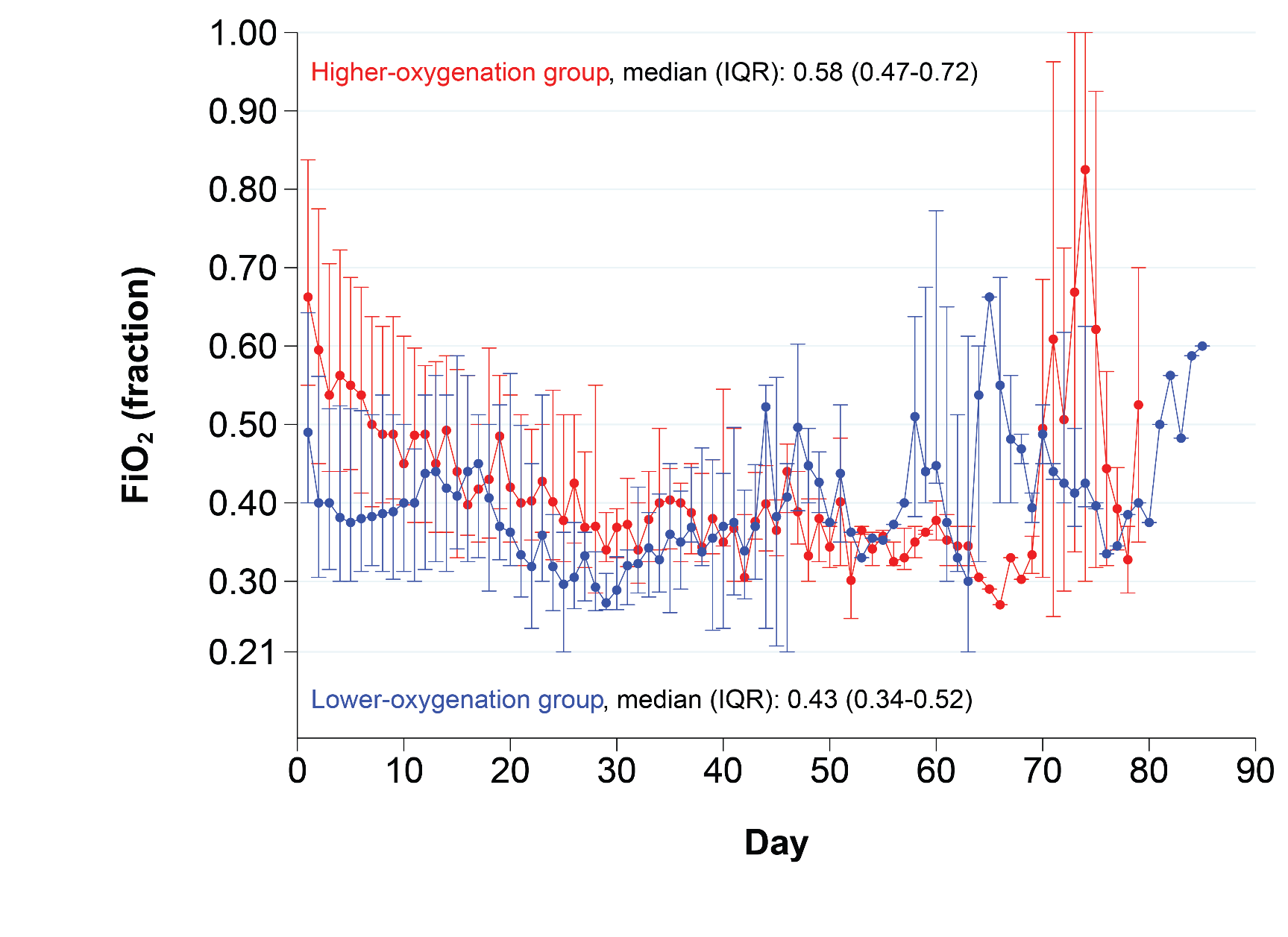
**

Median values of daily patient-means of fractions of inspired oxygen (FiO_2_) stratified according to oxygenation target allocation for the 90-day intervention period in patients with chronic obstructive pulmonary disease. Daily patient-means were calculated from the registered values corresponding to the 12-hour highest and lowest partial pressure of arterial oxygen. Bars represent interquartile ranges (IQR).

# **Figure S4. Time-weighted average PaO_2_**


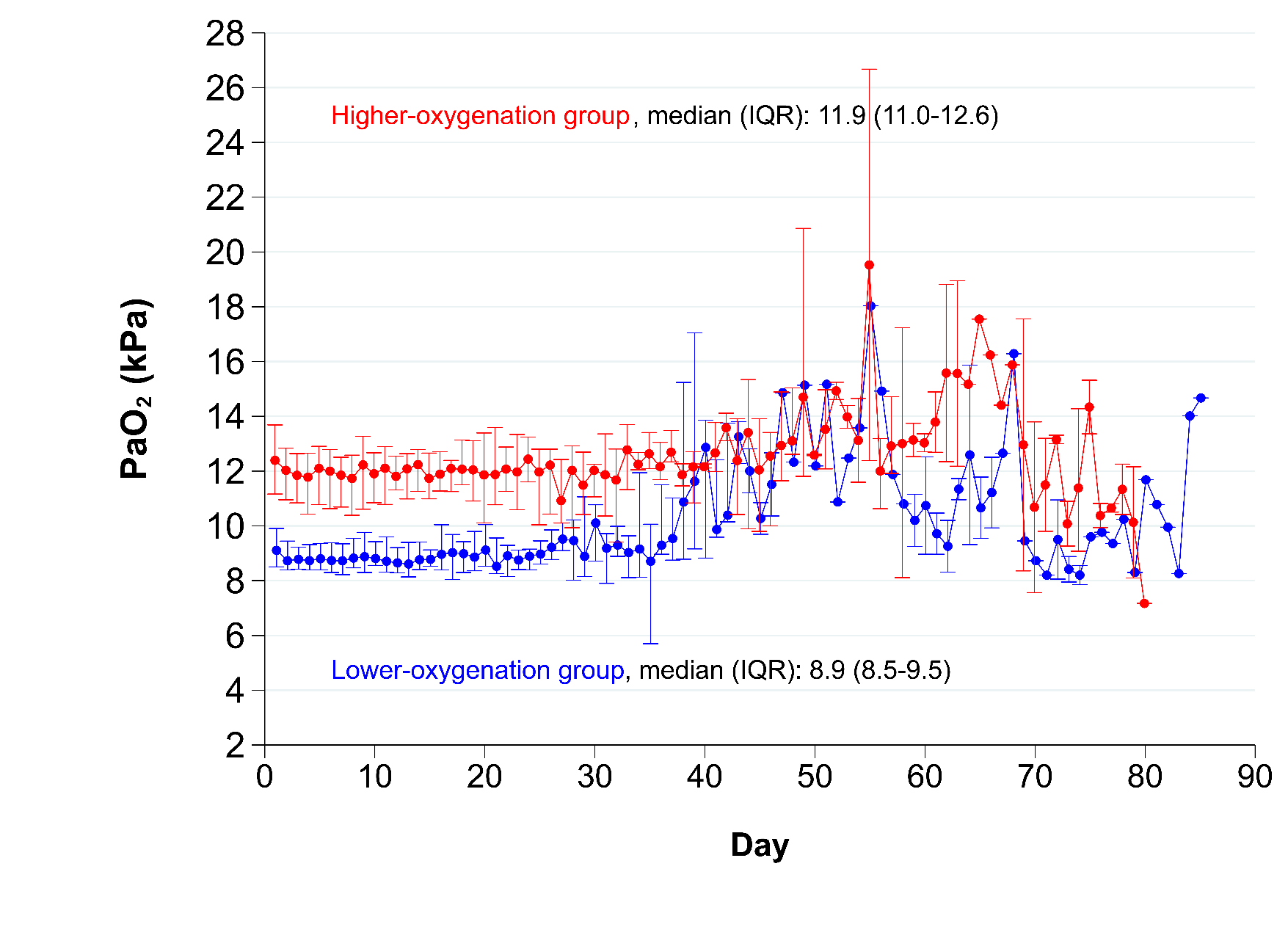


Daily medians of all patients’ time-weighted average partial pressures of arterial oxygen (PaO_2_) for the 90-day intervention period for the 497 Danish COPD patients (88.3% of all COPD patients included). Bars represent interquartile ranges (IQR).

# **Figure S5. Time-weighted average SaO_2_**


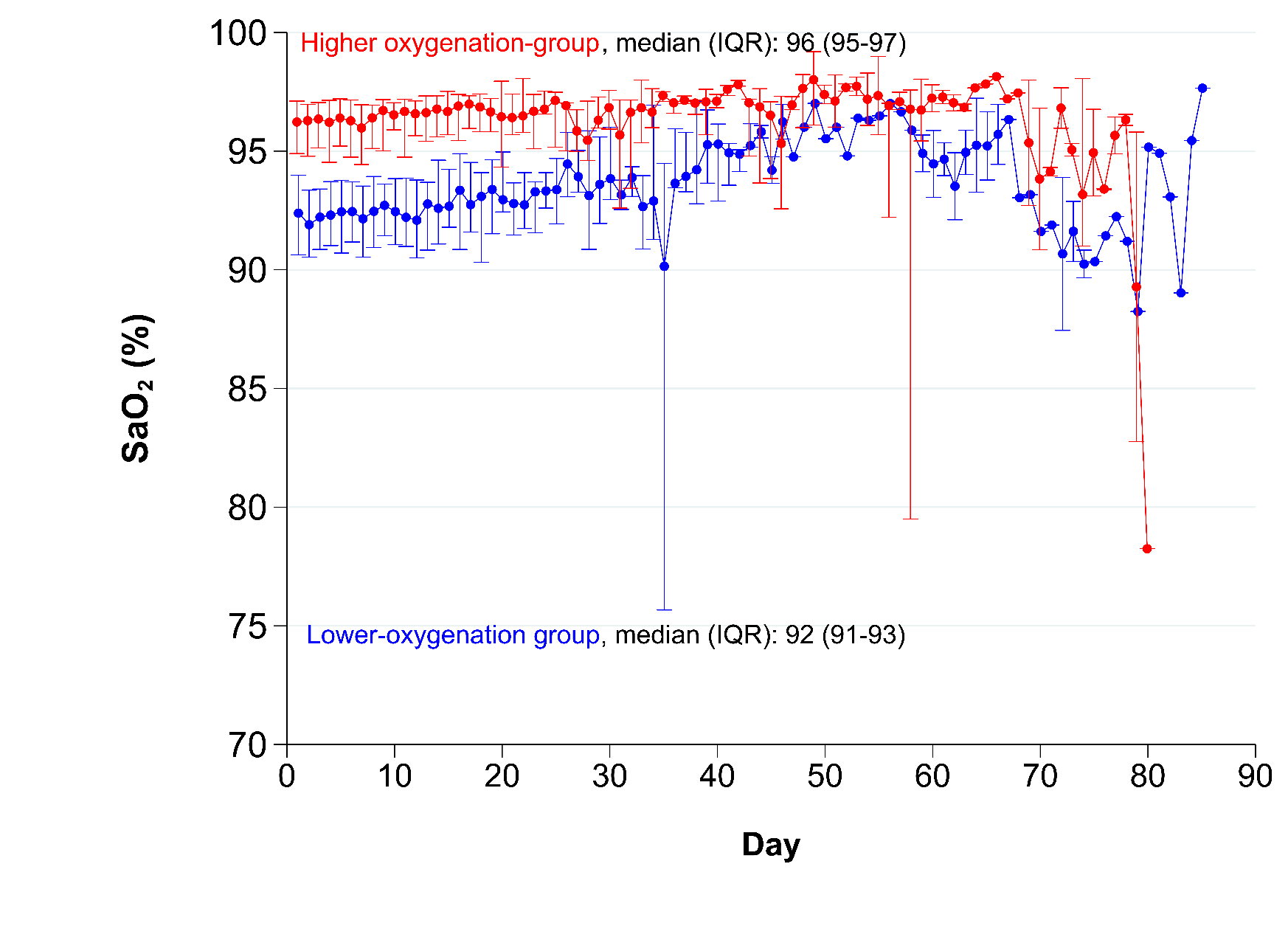


Daily medians of all patients’ time-weighted average oxygen saturation (SaO_2_) for the 90-day intervention period for the 497 Danish COPD patients (88.3% of all COPD patients included). Bars represent interquartile ranges (IQR).

# **Figure S6. Time-weighted average PaCO_2_**


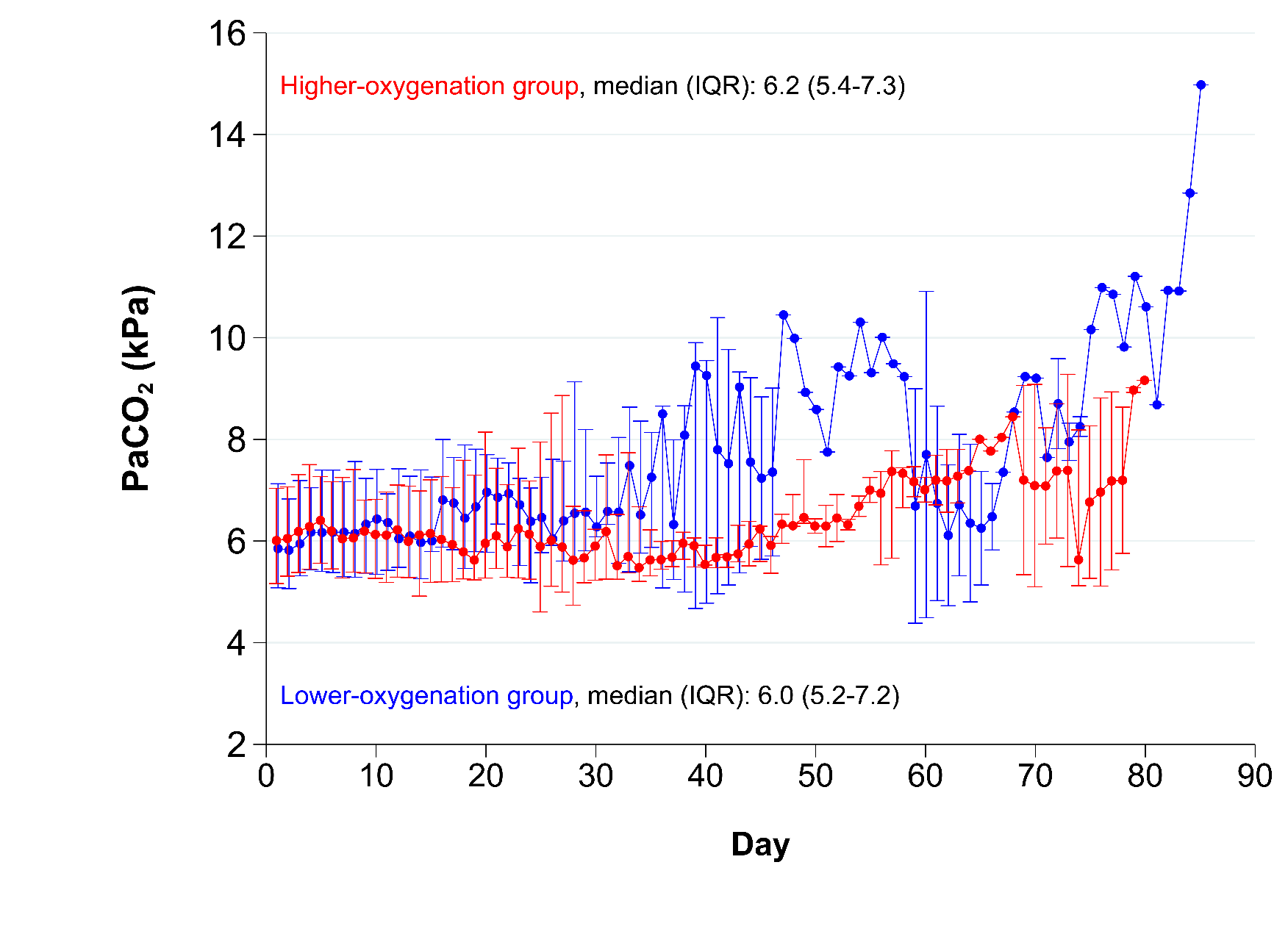


Daily medians of all patients’ time-weighted average partial pressure of arterial carbon dioxide (PaCO_2_) for the 90-day intervention period for the 497 Danish COPD patients (88.3% of all COPD patients included). Bars represent interquartile ranges (IQR).

# **Figure S7. Time-weighted average pH**


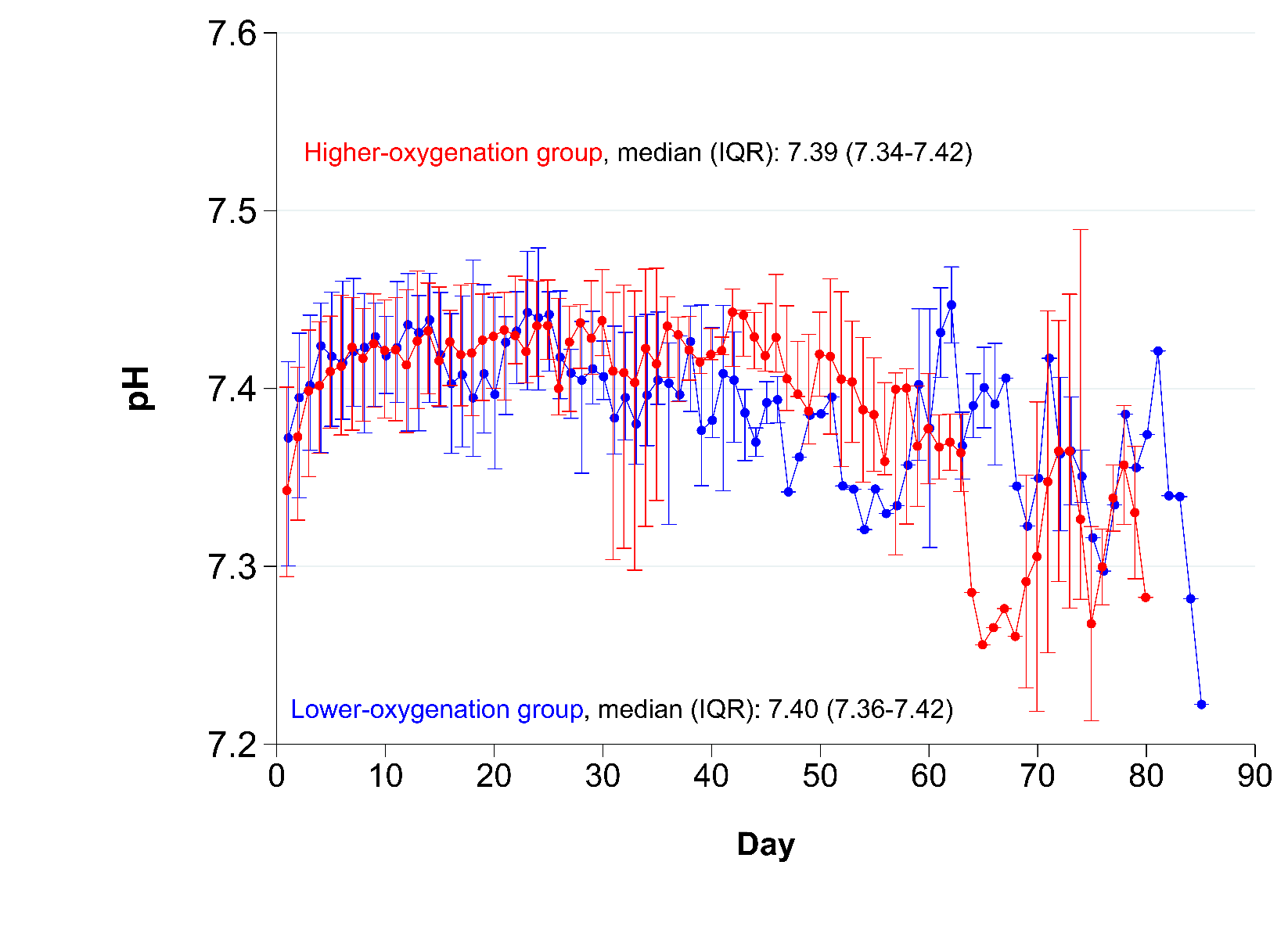


Daily medians of all patients’ time-weighted average pH for the 90-day intervention period for the 497 Danish COPD patients (88.3% of all COPD patients included). Bars represent interquartile ranges (IQR).

# **Figure S8. Time-weighted average SBC**


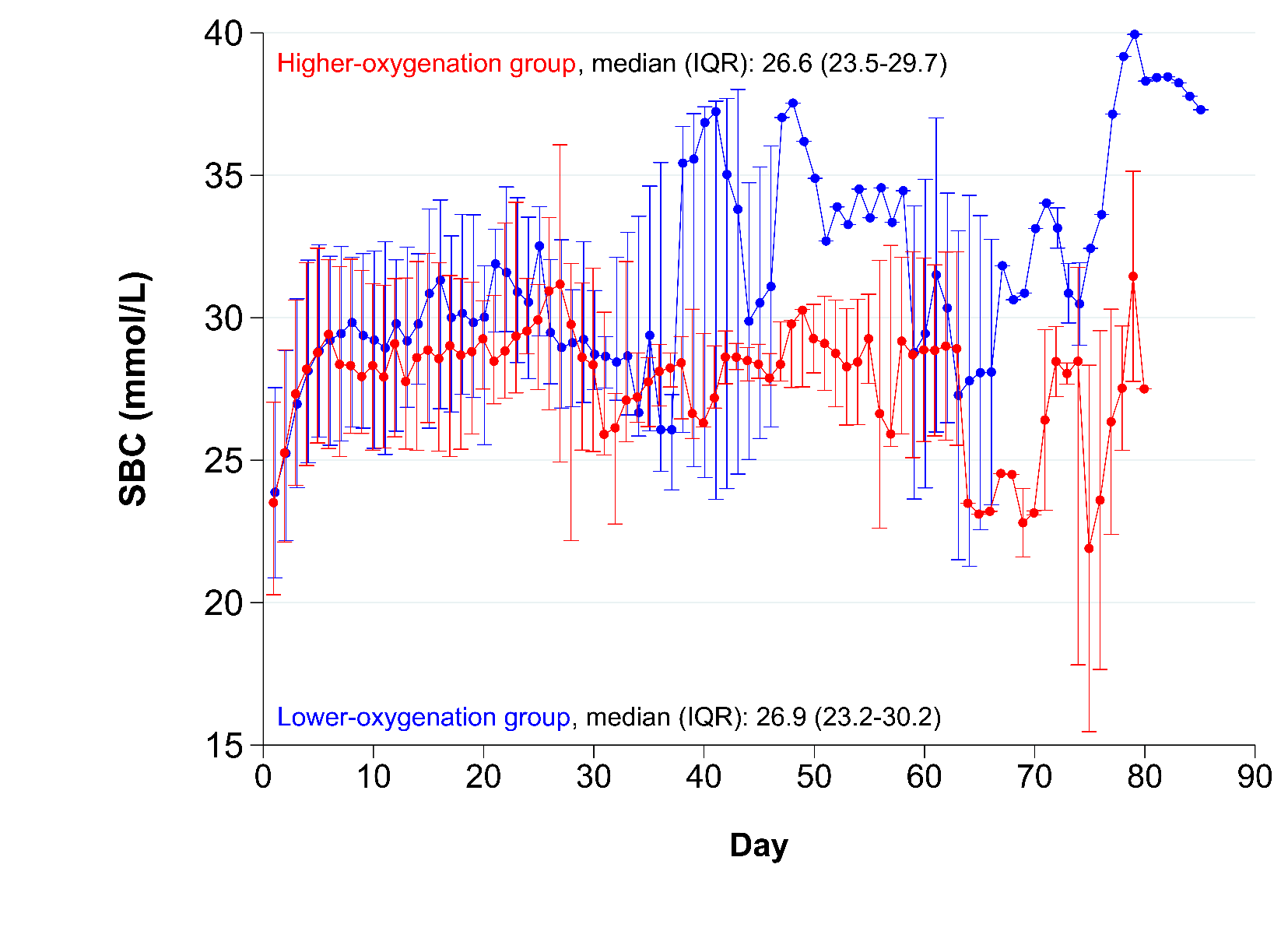


Daily medians of all patients’ time-weighted average standard bicarbonate concentration (SBC) for the 90-day intervention period for the 497 Danish COPD patients (88.3% of all COPD patients included). Bars represent interquartile ranges (IQR).

# Figure S9. Number of patients providing arterial blood gas data for the full arterial blood gas analyses


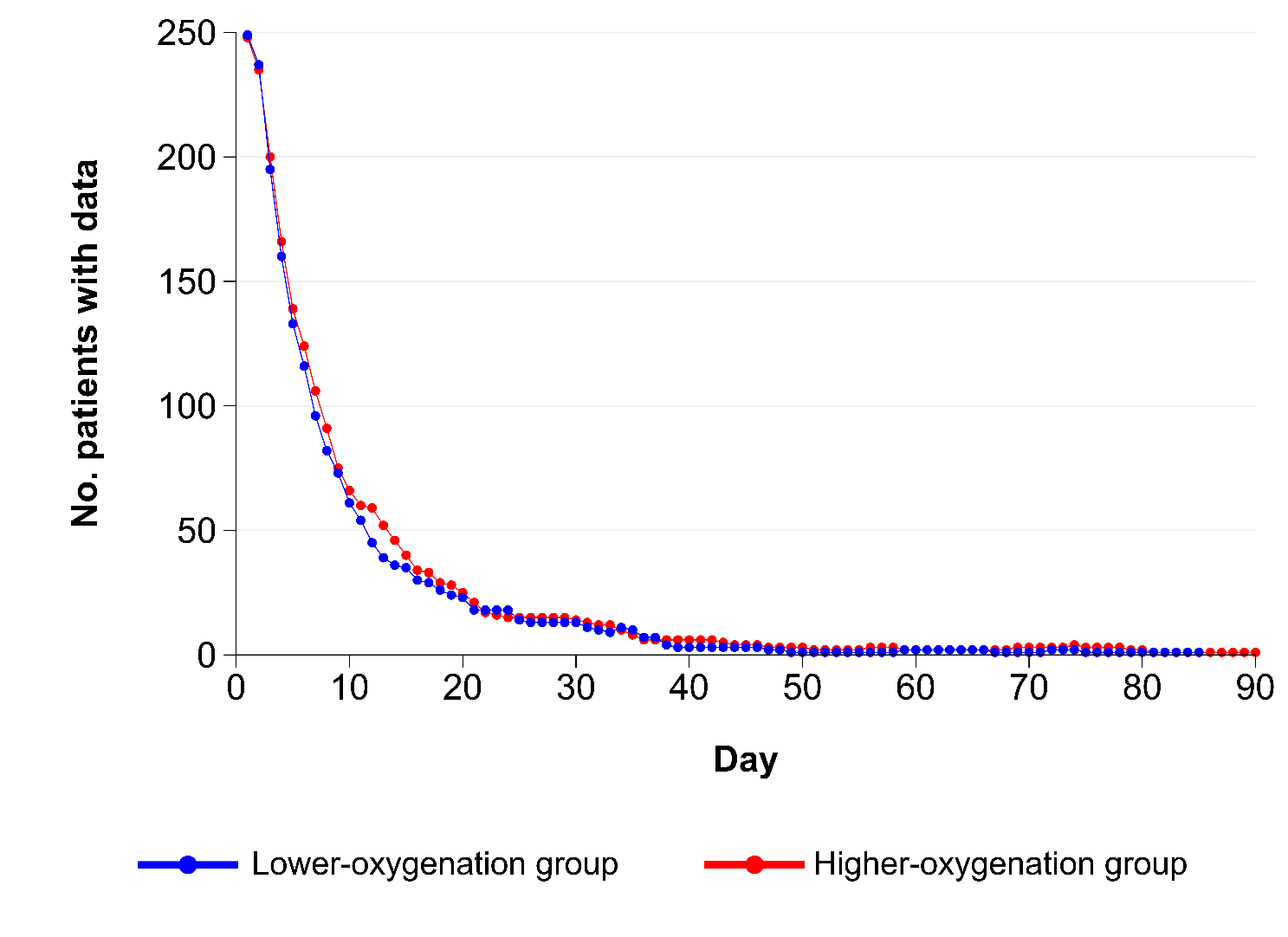


Number of Danish COPD patients (total n=497 (88.3% of all COPD patients included)) contributing with arterial blood gas data, per day.

# Figure S10. Number of arterial blood gases per day for the full arterial blood gas analyses


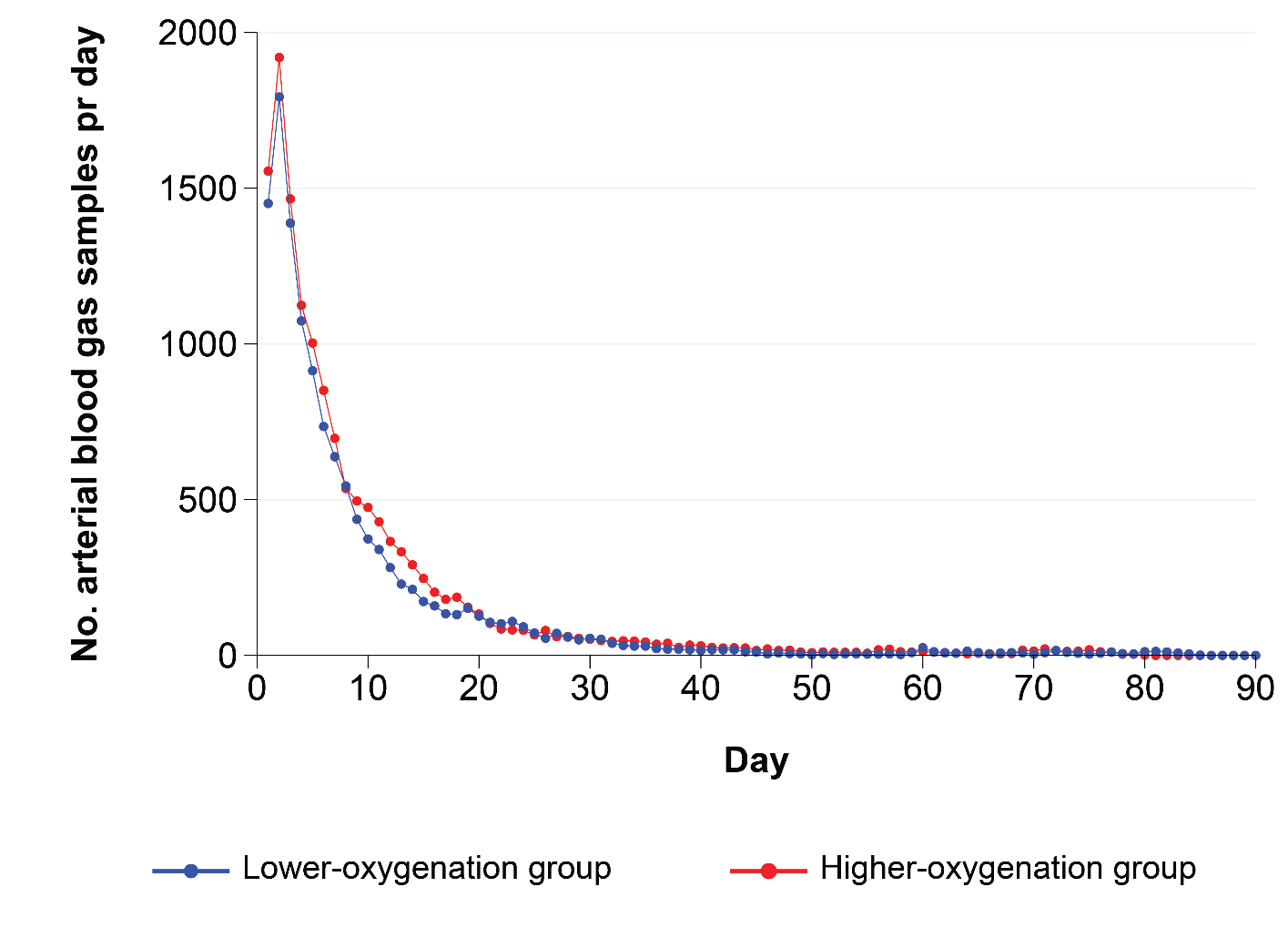


Total number of arterial blood gases per day for the 497 Danish COPD patients (88.3% of all COPD patients included).

# Table S1. Baseline Characteristics of patients with and without COPD

| **Characteristic** | **Patients with COPD**  **(n = 563)** | **Patients without COPD**  **(n = 2347)** |
| --- | --- | --- |
| **Age (IQR)** | 71 (64—77) | 69 (59—77) |
| **Male sex, n (%)** | 342 (60.8) | 1529 (65.2) |
| **Time from hospital admission to randomisation, days (IQR)** | 1 (0—4) | 1 (0—5) |
| **Time from ICU admission and randomisation, hours (IQR)** | 3.4 (1.4—7.0) | 3.6 (1.7—7.4) |
| **Coexisting illness, n (%)** |  |  |
| Ischaemic heart disease | 90 (16.0) | 320 (13.6) |
| Chronic heart failure | 85 (15.1) | 201 (8.6) |
| Active metastatic cancer | 17 (3.0) | 109 (4.6) |
| Long-term dialysis | 6 (1.1) | 41 (1.8) |
| Active haematologic malignancy | 16 (2.8) | 152 (6.5) |
| **Type of admission, n (%)** |  |  |
| Medical | 490 (87.0) | 1998 (85.1) |
| Elective surgery | 11 (2.0) | 28 (1.2) |
| Emergency surgery | 62 (11.0) | 321 (13.7) |
| **Acute illness, n (%)** |  |  |
| Pneumonia | 344 (61.1) | 1330 (56.7) |
| Multiple trauma | 8 (1.4) | 45 (1.9) |
| Haemorrhagic or ischaemic stroke | 6 (1.1) | 41 (1.8) |
| Traumatic brain injury | 3 (0.5) | 21 (0.9) |
| Myocardial infarction | 30 (5.3) | 153 (6.5) |
| Intestinal ischaemia | 11 (2.0) | 57 (2.4) |
| Cardiac arrest | 56 (10.0) | 279 (11.9) |
| ARDS | 44 (7.8) | 329 (14.0) |
| **Invasive ventilation** |  |  |
| Patients, n (%) | 327 (58.1) | 1377 (58.7) |
| Tidal volume, ml (IQR) | 501 (425—578) | 498 (429—573) |
| End-expiratory pressure, cm H_2_O (IQR) | 8 (7—10) | 10 (7—10) |
| Peak pressure, cm H_2_O (IQR) | 26 (21—30) | 25 (20—29) |
| **Non-invasive ventilation or CPAP** |  |  |
| Patients, n (%) | 104 (18.5) | 271 (11.6) |
| End-expiratory pressure, cm H_2_O (IQR) | 7 (5—8) | 7 (5—9) |
| **Open system, n (%)** | 132 (23.5) | 699 (29.8) |
| **PaO_2_, kPa (IQR)** | 10.4 (8.7–12.8) | 10.3 (8.7–12.4) |
| **SaO_2_, % (IQR)*** | 94 (91—97) | 95 (91—97) |
| **PaCO_2_, kPa (IQR)^†^** | 6.4 (5.4—8.3) | 5.5 (4.7—6.7) |
| **pH (IQR)^†^** | 7.31 (7.24—7.37) | 7.34 (7.26—7.42) |
| **SBC, mmol L^-1^ (IQR)^†^** | 22.5 (19.7—26.0) | 22.0 (18.8—25.1) |
| **FiO_2_, fraction (IQR)^‡^** | 0.62 (0.55—0.80) | 0.70 (0.58—0.90) |
| **PaO_2_:FiO_2_ ratio, kPa (IQR)** |  |  |
| In all systems | 16.2 (12.9—21.4) | 15.6 (11.6—20.6) |
| Invasive ventilation | 17.8 (13.6—22.7) | 16.5 (12.2—21.8) |
| NIV/CPAP | 16.4 (13.5—20.8) | 15.6 (11.8—20.2) |
| Open systems | 13.9 (11.6—18.0) | 14.1 (10.7—18.3) |
| **Lactate concentration, mmol L^-1^ (IQR)** | 1.6 (1.0—2.8) | 1.8 (1.1—3.3) |
| **Lowest mean arterial pressure, mmHg (IQR)** | 58 (49—68) | 58 (48—68) |
| **Use of inotropes, n (%)** | 9 (1.6) | 61 (2.6) |
| **Use of vasopressors** |  |  |
| Patients, n (%) | 293 (52.0) | 1298 (55.3) |
| Highest dose of NE, μg kg^-1^ min^-1^ (IQR)^§^ | 0.2 (0.1—0.4) | 0.2 (0.1—0.4) |
| **SOFA score (IQR)^¶^** | 7 (5—9) | 8 (5—10) |

IQR = interquartile range. ICU = intensive care unit. ARDS = acute respiratory distress syndrome. CPAP = continuous positive airway pressure. PaO_2_ = partial pressure of arterial oxygen. SaO_2_ = oxygen saturation. PaCO_2_ = partial pressure of arterial carbon dioxide. SBC = standard bicarbonate. FiO_2_ = fraction of inspired oxygen. NIV = non-invasive ventilation. NE = norepinephrine. SOFA = Sequential Organ Failure Assessment.

Values are medians unless stated otherwise.

* Values for SaO_2_ were missing for 25 patients with COPD and 166 without COPD due to this analysis being unavailable at one

trial site

^†^ PaCO_2_, pH and SBC values are based on arterial blood gases from the 497 and 1835 Danish patients included in the HOT-ICU

trial with and without COPD, respectively

^‡^ FiO_2_ in open systems estimated using standardised conversion tables (Table S4)

^§^ In patients receiving norepinephrine

^¶^ SOFA scores range from 0–24, with higher scores indicating more severe organ failure

# Table S2. Intensive care unit treatment parameters in patients with COPD

| **Variable** | **Lower-oxygenation group**  **(n = 277)** | **Higher-oxygenation group**  **(n = 286)** |
| --- | --- | --- |
| **Mean number of daily arterial blood gas samples (SD)** | 6 (2) | 6 (2) |
| **Number of patients receiving mechanical ventilation*** |  |  |
| Any use of IMV, n (%) | 201 (72.6) | 206 (72.0) |
| Any use of NIV or CPAP, n (%) | 84 (30.3) | 80 (28.0) |
| **Respiratory support*** |  |  |
| Median number of days on IMV, all patients (IQR) | 2 (0–6) | 3 (0–7) |
| Median number of days on IMV in patients receiving IMV at any time (IQR) | 4 (2–8) | 4 (2–10) |
| Median number of days on NIV/CPAP, all patients (IQR) | 0 (0–1) | 0 (0–1) |
| Median number of days on NIV/CPAP in patients receiving NIV/CPAP at any time   (IQR) | 1 (1–2) | 1 (1–2) |
| **Any mechanical ventilation in prone position, n (%)** | 8 (2.9) | 10 (3.5) |
| **Any use of inhaled vasodilators, n (%)** | 6 (2.2) | 14 (4.9) |
| **Any use of ECMO, n (%)** | 1 (0.4) | 1 (0.4) |
| **Any blood transfusions, n (%)** | 58 (21.0) | 75 (26.2) |
| Median number of blood transfusions (IQR) | 2 (1–4) | 2 (1–5) |
| Median transfused volume (IQR), ml | 600 (300–980) | 600 (300–1225) |
| **Invasive ventilation*** |  |  |
| Median PEEP (IQR), cm H_2_O | 8 (7–10) | 9 (8–10) |
| Median TV kg^-1^ (IQR), ml/kg | 7.4 (6.5–8.2) | 7.5 (6.6–8.4) |
| Median peak pressure (IQR), cm H_2_O | 22 (19–26) | 22 (19–27) |
| **NIV or CPAP*** |  |  |
| Median EPAP / CPAP (IQR), cm H_2_O | 7 (6–8) | 8 (6–10) |
| **Any use of vasopressors or inotropes, n (%)** | 211 (76.2) | 235 (82.2) |
| **Any use of renal replacement therapy, n (%)** | 32 (11.6) | 38 (13.3) |

SD = standard deviation. IMV = invasive mechanical ventilation. NIV = non-invasive ventilation. CPAP = continuous positive airway pressure. IQR = interquartile range. ECMO = extra corporal membrane oxygenation. PEEP = positive end-expiratory pressure. TV = tidal volume. EPAP = end-expiratory pressure.

* Ventilation parameters registered once daily at 08:00 hours

# Table S3. Intensive care unit treatment parameters in patients with and without COPD

| **Variable** | **Patients with COPD**  **(n = 563)** | **Patients without COPD (n = 2347)** |
| --- | --- | --- |
| **Mean number of daily arterial blood gas samples (SD)** | 6 (2.2) | 6 (2.3) |
| **Number of patients receiving mechanical ventilation*** |  |  |
| Any use of IMV, n (%) | 407 (72.3) | 1805 (76.9) |
| Any use of NIV or CPAP, n (%) | 164 (29.1) | 485 (20.7) |
| **Respiratory support*** |  |  |
| Median number of days on IMV, all patients (IQR) | 2 (0–6) | 3 (1–8) |
| Median number of days on IMV in patients receiving IMV at any time (IQR) | 4 (2–9) | 5 (2–11) |
| Median number of days on NIV/CPAP (IQR) | 0 (0–1) | 0 (0–0) |
| Median number of days on NIV/CPAP in patients receiving NIV/CPAP at any time  (IQR) | 1 (1–2) | 1 (1–2) |
| **Any mechanical ventilation in prone position, n (%)** | 18 (3.2) | 149 (6.4) |
| **Any use of inhaled vasodilators, n (%)** | 20 (3.6) | 104 (4.4) |
| **Any use of ECMO, n (%)** | 2 (0.4) | 24 (1.0) |
| **Any blood transfusions, n (%)** | 133 (23.6) | 821 (35.0) |
| Median number of blood transfusions (IQR) | 2 (1–4) | 3 (1–6) |
| Median transfused volume (IQR), ml | 600 (300–1200) | 735 (300–1500) |
| **Invasive ventilation*** |  |  |
| Median PEEP (IQR), cmH_2_O | 8 (7–10) | 9 (8–10) |
| Median TV kg^-1^ (IQR), ml/kg | 7.5 (6.5–8.3) | 7.3 (6.4–8.2) |
| Median peak pressure (IQR), cm H_2_O | 22 (19–27) | 22 (18–26) |
| **Non-invasive ventilation or CPAP*** |  |  |
| Median EPAP / CPAP (IQR), cmH_2_O | 8 (6–8) | 8 (6–9) |
| **Any use of vasopressors or inotropes, n (%)** | 446 (79.2) | 1913 (81.5) |
| **Any use of renal replacement therapy, n (%)** | 70 (12.4) | 533 (22.7) |

SD = standard deviation. IMV = invasive mechanical ventilation. NIV = non-invasive ventilation. CPAP = continuous positive airway pressure. IQR = interquartile range. ECMO = extra corporal membrane oxygenation. PEEP = positive end-expiratory pressure. TV = tidal volume. EPAP = end-expiratory pressure.

* Ventilation parameters registered once daily at 08:00 hours

# Table S4. Number of COPD patients providing data on oxygenation in the HOT-ICU trial

|  | **PaO_2_** | | **FiO_2_** | | **SaO_2_** | |
| --- | --- | --- | --- | --- | --- | --- |
| **Day** | **Lower-oxygenation**  **group** | **Higher-oxygenation**  **group** | **Lower-oxygenation**  **group** | **Higher-oxygenation**  **group** | **Lower-oxygenation**  **group** | **Higher-oxygenation**  **group** |
| ***1*** | 271 | 283 | 271 | 283 | 260 | 269 |
| ***10*** | 61 | 79 | 61 | 79 | 58 | 71 |
| ***20*** | 23 | 29 | 23 | 29 | 20 | 26 |
| ***30*** | 12 | 14 | 12 | 14 | 11 | 12 |
| ***40*** | 5 | 5 | 5 | 5 | 4 | 5 |
| ***50*** | 1 | 2 | 1 | 2 | 1 | 2 |
| ***60*** | 3 | 2 | 3 | 2 | 2 | 2 |
| ***70*** | 2 | 2 | 2 | 2 | 1 | 2 |
| ***80*** | 1 | 0 | 1 | 0 | 1 | 0 |
| ***90*** | 0 | 0 | 0 | 0 | 0 | 0 |

COPD = chronic obstructive pulmonary disease. PaO_2_ = partial pressure of arterial oxygen. FiO_2_ = fraction of inspired oxygen. SaO_2_ = arterial oxygen saturation.

Number of patients with data on oxygenation parameters stratified by treatment allocation. Data on SaO_2_ is missing for 25 patients as values were not available from one site.

# Table S5. Fraction of inspired oxygen (FiO_2_) conversion tables for open systems

| **Nasal cannula**: flow of  oxygen and corresponding **FiO_2_**  0 L/min: **0.21**  1 L/min: **0.27**  2 L/min: **0.33**  3 L/min: **0.37**  4 L/min: **0.40**  5 L/min: **0.44**  6 L/min: **0.48**  10 L/min: **0.62** |
| --- |

| **Hudson masks or similar:**  Flow of oxygen and corresponding **FiO_2_**  6 L/min: **0.45**  8 L/min: **0.50**  10 L/min: **0.54**  15 L/min: **0.59**  30 L/min: **0.65** |
| --- |

| **Hudson mask or similar**, when using air/oxygen mixtures**:**  Flow of oxygen/air and corresponding **FiO_2_**  3 L O_2_ / 12 L air /min (≈ 37%): **0.29**  7.5 L O_2_ / 7.5 L air /min (≈ 60%): **0.41**  10 L O_2_ / 5 L air /min (≈ 74%): **0.48**  12 L O_2_ / 18 L air /min (≈ 52%): **0.39** |
| --- |

| If a **Venturi-mask** is used, use the **FiO_2_** as  stated on the respective mask (colour code), typical range **0.24** to **0.60** |
| --- |

| If **high flow humidified oxygen via nasal cannula** **≥ 15 L/min** is used:  The FiO_2_ equals the oxygen concentration as stated on the mixer **(0.21 to 1.00)** |
| --- |

| **Reservoir-masks (non-rebreather masks)** with flows ≥ 10 L/min, **FiO_2_** = **0.95** |
| --- |

Conversion tables were pragmatically adapted from Waldau et al,^4^ Boumphrey et al,^5^ and Chanques et al.^6^

#
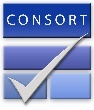
CONSORT 2010 checklist

| Section/Topic | Item No | Checklist item | Reported on page No |
| --- | --- | --- | --- |
| Title and abstract | | | |
|  | 1a | Identification as a randomised trial in the title | 1 |
|  | 1b | Structured summary of trial design, methods, results, and conclusions (for specific guidance see CONSORT for abstracts) | 2 |
| Introduction | | | |
| Background and objectives | 2a | Scientific background and explanation of rationale | 5–6 |
|  | 2b | Specific objectives or hypotheses | 5–6 |
| Methods | | | |
| Trial design | 3a | Description of trial design (such as parallel, factorial) including allocation ratio | 7 |
|  | 3b | Important changes to methods after trial commencement (such as eligibility criteria), with reasons | Details are provided in primary publication^1^ |
| Participants | 4a | Eligibility criteria for participants | 7 and Supplementary appendix |
|  | 4b | Settings and locations where the data were collected | 7 |
| Interventions | 5 | The interventions for each group with sufficient details to allow replication, including how and when they were actually administered | 8 |
| Outcomes | 6a | Completely defined pre-specified primary and secondary outcome measures, including how and when they were assessed | 8 + Supplementary appendix |
|  | 6b | Any changes to trial outcomes after the trial commenced, with reasons | Details are provided in primary publication^1^ |
| Sample size | 7a | How sample size was determined | Details are provided in primary publication^1^ |
|  | 7b | When applicable, explanation of any interim analyses and stopping guidelines | Details are provided in primary publication^1^ |
| Randomisation: |  |  |  |
| Sequence generation | 8a | Method used to generate the random allocation sequence | 7–8 |
|  | 8b | Type of randomisation; details of any restriction (such as blocking and block size) | 7–8 |
| Allocation concealment mechanism | 9 | Mechanism used to implement the random allocation sequence (such as sequentially numbered containers), describing any steps taken to conceal the sequence until interventions were assigned | 7–8 |
| Implementation | 10 | Who generated the random allocation sequence, who enrolled participants, and who assigned participants to interventions | 7–8 |
| Blinding | 11a | If done, who was blinded after assignment to interventions (for example, participants, care providers, those assessing outcomes) and how | Details are provided in primary publication^1^ |
|  | 11b | If relevant, description of the similarity of interventions | NA |
| Statistical methods | 12a | Statistical methods used to compare groups for primary and secondary outcomes | 8–9 |
|  | 12b | Methods for additional analyses, such as subgroup analyses and adjusted analyses | 8–9 |
| Results | | | |
| Participant flow (a diagram is strongly recommended) | 13a | For each group, the numbers of participants who were randomly assigned, received intended treatment, and were analysed for the primary outcome | 10 + Fig 1 |
|  | 13b | For each group, losses and exclusions after randomisation, together with reasons | 10 + Fig 1 |
| Recruitment | 14a | Dates defining the periods of recruitment and follow-up | 10 |
|  | 14b | Why the trial ended or was stopped | Inclusion of pre-specified number of patients |
| Baseline data | 15 | A table showing baseline demographic and clinical characteristics for each group | Table 1 |
| Numbers analysed | 16 | For each group, number of participants (denominator) included in each analysis and whether the analysis was by original assigned groups | 10–11 + Table 2 |
| Outcomes and estimation | 17a | For each primary and secondary outcome, results for each group, and the estimated effect size and its precision (such as 95% confidence interval) | 10–11 + Table 2 |
|  | 17b | For binary outcomes, presentation of both absolute and relative effect sizes is recommended | 10–11 + Table 2 |
| Ancillary analyses | 18 | Results of any other analyses performed, including subgroup analyses and adjusted analyses, distinguishing pre-specified from exploratory | 10–11 + Table 2 |
| Harms | 19 | All important harms or unintended effects in each group | 10–11 + Table 2 |
| Discussion | | | |
| Limitations | 20 | Trial limitations, addressing sources of potential bias, imprecision, and, if relevant, multiplicity of analyses | 13–14 |
| Generalisability | 21 | Generalisability (external validity, applicability) of the trial findings | 12–14 |
| Interpretation | 22 | Interpretation consistent with results, balancing benefits and harms, and considering other relevant evidence | 14 |
| Other information | | |  |
| Registration | 23 | Registration number and name of trial registry | 3 |
| Protocol | 24 | Where the full trial protocol can be accessed, if available | Protocol and statistical analysis plan published online.^2, 3^ |
| Funding | 25 | Sources of funding and other support (such as supply of drugs), role of funders | 15 |

# References

1. Schjørring OL, Klitgaard TL, Perner A, et al. Lower or Higher Oxygenation Targets for Acute Hypoxemic Respiratory Failure. *New England Journal of Medicine* 2021; **384**: 1301–11

2. Schjørring OL, Perner A, Wetterslev J, et al. Handling Oxygenation Targets in the Intensive Care Unit (HOT‐ICU)—Protocol for a randomised clinical trial comparing a lower vs a higher oxygenation target in adults with acute hypoxaemic respiratory failure. *Acta Anaesthesiol Scand* 2019; aas.13356

3. Schjørring OL, Klitgaard TL, Perner A, et al. The Handling Oxygenation Targets in the Intensive Care Unit (HOT-ICU) trial: Detailed statistical analysis plan. *Acta Anaesthesiol Scand* [Internet] 2020; **64**: 847–56 Available from: <http://www.ncbi.nlm.nih.gov/pubmed/32068884>

4. Waldau T, Larsen VH, Bonde J. Evaluation of five oxygen delivery devices in spontaneously breathing subjects by oxygraphy. *Anaesthesia* 1998; **53**: 256–63

5. Boumphrey SM, Morris EAJ, Kinsella SM. 100% inspired oxygen from a Hudson mask-a realistic goal!; Resuscitation 2003.pdf. *Resuscitation* 2003; **57**: 69–72

6. Chanques G, Riboulet F, Molinari N, et al. Comparison of three high flow oxygen therapy delivery devices: A clinical physiological cross-over study. *Minerva Anestesiol* 2013; **79**: 1344–55
